# Using submerged fermentation to fast increase N6-(2-hydroxyethyl)-adenosine, adenosine and polysaccharide productions of *Cordyceps cicadae* NTTU 868

**DOI:** 10.1186/s13568-019-0892-4

**Published:** 2019-12-09

**Authors:** Bo-Jun Ke, Chun-Lin Lee

**Affiliations:** Department of Life Science, National Taitung University, 369, Sec. 2, University Rd., Taitung, 95092 Taiwan, ROC

**Keywords:** *Cordyceps cicadae* NTTU 868, Adenosine, N6-(2-hydroxyethyl)-adenosine (HEA), Polysaccharide

## Abstract

*Cordyceps cicadae* is a well-known traditional Chinese medicine for treating palpitations and eye diseases. It contains several bioactive compounds such as adenosine, N6-(2-hydroxyethyl)-adenosine (HEA), and polysaccharide. Those bioactive compounds have been reported to perform anti-oxidation and anti-inflammatory properties and provide renal protection. In this study, we researched different fermentation conditions in order to enhance the biomass, adenosine, HEA, and polysaccharide productions of *C. cicadae* NTTU 868. Solid fermentation was carried out with different grain substrates (barley, oat, rice and wheat). Various submerged fermentation scales were used to produce the *C. cicadae* NTTU 868 mycelium. The results of solid fermentation revealed that *C. cicadae* NTTU 868 produced higher adenosine and HEA concentrations in oat rather than in other substrates. *C. cicadae* NTTU 868 mycelium had obtained the highest concentrations of adenosine and HEA on Day 2 as using the small-scale submerged fermentation. Furthermore, potato dextrose broth with extra 0.2% of yeast extract was able to result in higher HEA concentration. In conclusion, using submerged fermentation to culture *C. cicadae* NTTU 868 resulted in more efficient adenosine, HEA, and polysaccharide productions than using solid-fermentation, especially when 0.2% of yeast extract was used in the PDB. Importantly, this can be easily scaled-up in the fermentation industry.

## Introduction

*Cordyceps* species are used in many pharmacological treatments including to treat cancer (Lee et al. 2006; Park et al. 2005), anti-oxidation (Yu et al. 2006), anti-inflammatory treatments (Lu et al. 2015; Won and Park 2005), anti-aging (Ramesh et al. 2012), immunopotentiation (Weng et al. 2002; Zhou et al. 2008) and hyperlipidemia (Sohn et al. 2012). However, *Cordycpes cicadae* is a well-known source of traditional Chinese *Cordycpes* medicine in Asia. The *Cicada flammata* worm and other insects are infected by fungi *C. cicadae* in winter. The fruit bodies grow on the pupa of the worm in summer. The length is about 0.1 to 4 cm. The wild *C. cicadae* grows in high humid conditions on mountains between 80 and 500 m high in Asia. Previous studies indicated that *C. cicadae* produced many bioactive compounds, such as adenosine (Wang et al. 2012; Zeng et al. 2014), N6-(2-hydroxyethyl)-adenosine (HEA) (Meng et al. 2015), ergosterol (Weng et al. 2002; Zhu et al. 2014), and polysaccharides (Sharma et al. 2018). In addition, HEA, one of the characteristic bioactive compounds in *C. cicadae,* has been proven to perform renal protective qualities, and anti-inflammatory property (Lu et al. 2015). *C. cicadae*-produced polysaccharide can inhibit the levels of reactive oxygen species (ROS) and exhibited the activities of anti-oxidative enzymes (Peng et al. 2013). Adenosine, a purine nucleoside also has anti-inflammatory and anti- hepatic fibrosis qualities and prevents diabetes (Koupenova and Ravid 2013).

*Cordyceps cicadae* fruit body has been harvested successfully produced using artificial solid culture but the culture is more than 50 days (Wang et al. 2014). To increase the functional compounds production and short the culture time were important topic for the development of *C. cicadae*. Submerged culture was another culture technology for the mycelium of *C. cicadae*. In our previous study, the submerged culture product of *C. cicadae* has been proven to prevent the development of liver-fibrosis in mice induced by CCl_4_ injection. Furthermore, HEA, adenosine, and polysaccharides produced by submerged culture were proven as the functional components for liver protection (Ke and Lee 2018). Therefore, submerged culture may be the novel culture to instead of solid fermentation for producing HEA and polysaccharides.

In this study, we researched different culture methods and conditions to short the culture time and produce high adenosine, HEA, and polysaccharide contents in *C. cicadae* NTTU 868. Furthermore, we also conducted pilot-up fermentation to create medical potential for the fermentation industry.

## Materials and methods

### Chemicals

99% purity of adenosine (Sigma-Aldrich Co., St. Louis, Missouri, USA) and 95% purity of HEA (Ark Pharm, Inc., Libertyville, Illinois, USA) were used as standard in the HPLC test.

### Isolation and culture

*Cordyceps cicadae* NTTU 868 (*C. cicadae* BCRC 930197) deposited in a Bioresource Collection and Resource Center (BCRC, Hsinchu, Taiwan) was incubated in potato dextrose agar (PDA) plates at 24 °C. Sub-culturing was done at 30-days intervals to maintain the viability. The seed culture was cultured in potato dextrose broth (PDB) using a 3-baffles Hinton flask with 100 rpm at 24 °C for 72 h.

### Solid fermentation

Barley, oat, rice and wheat were used as grain substrates. 100 g of grain substrates were mixed with PDA in plastic boxes, which had 14 cotton balls acting as an air tunnel which were sterilized in an autoclave at 121 °C, 1.5 atm pressure for 30 min. 10% of the seed-cultured solution was added into the grain substrates. In the first 10 days, the solid fermentation was incubated at 24 °C without light irradiation to let the mycelium grow in the grain substrates. 10 days later, mycelium covered the grain substrates entirely and the base of the fruit body appeared on the surface. Then the solid fermentation was incubated at 20 °C for 50 days with light irradiation to irritate fruit body production. The *C. cicadae* NTTU 868 fruit bodies were collected and dried at 55 °C.

### Submerged fermentation

On alternate days submerged fermentation was cultured in PDB with a 10% seed-cultured solution using a 3-baffles shake flask at 24 °C, shaking at 100 rpm on the 2nd, 4th, 6th, 8th, 10th and 12th day. 5% of different grain powders (barley, oat or rice) also were used in the submerged fermentation and incubated for 2 and then 10 days. Furthermore, carbon (dextrose, mannitol, or sucrose), nitrogen (monosodium glutamate, peptone or yeast extract) and mineral (potassium chloride, magnesium chloride or ferric chloride) supplements were also used in the submerged fermentation and incubated for 10 days. *C. cicadae* NTTU 868 mycelium was collected and lyophilized.

### Pilot-up submerged fermentation

The 2-L bottle including 1.5-L of PDB with an additional 0.2% of yeast extract was designed and manufactured to be a fermentor with a culture condition of 24 °C and 1.5 v/v/m aeration. The *C. cicadae* NTTU 868 mycelium was sampled once every 2 days and then lyophilized for analysis.

### Extraction and quantitative analysis of adenosine and HEA

To extract adenosine, and HEA, *C. cicadae* NTTU 868 fruit body and mycelium were incubated with 10 volumes of 20% methanol at 60 °C for 30 min and vortexed at every 10 min intervals. Then, we collected the *C. cicadae* NTTU 868 fruit body and mycelium extract by centrifugation at 15,000 rpm for 10 min. Next, the extraction was incubated at − 20 °C overnight and then centrifuged at 15,000 rpm for 10 min to remove polysaccharide (Hung et al. 2015). The supernatant was collected.

Adenosine and HEA were determined by high performance liquid chromatography (HPLC) with a reverse-phase column (Mightysil RP-18 GP 5 μm C18, 250–4.6 mm, Kanto Chemical Co., Inc., Tokyo, Japan) and diode array detector (DAD, L-2000 series, Hitachi, Japan). The mobile phase (A solvent: methanol; B solvent: water) was eluted with 0.8 mL/min of flow rate and gradient condition (A solvent: methanol; B solvent: water; 0–3 min, 10% A; 4–8 min, 0% A; 8–18 min, 0% A to 100% A; 18–25 min, 10% A, 28 to 30 min). 20 μL sample was injected each time. Absorption spectra of eluted compounds were recorded at 262 nm (Lu et al. 2015).

### Extraction and quantitative analysis of polysaccharide

*Cordyceps cicadae* NTTU 868 polysaccharide were extracted by 10 volumes of ultra-pure water at 95 °C for 60 min and vortexed at 10 min intervals. The supernatant was collected by centrifugation at 15,000 rpm for 10 min. The extract was mixed with 5 volumes of 95% ethanol and then incubated at 4 °C overnight. The extract was then centrifuged at 15,000 rpm for 10 min and collected using insoluble precipitate (polysaccharide). To quantify the concentration of polysaccharide, we conducted phenol–sulfuric acid assay.

### Statistics

The data is presented as the mean ± SD. The means followed by a different letter within each column have significant difference from each other (one-way analysis of variance (ANOVA) with Duncan’s multiple rang test, *p* < 0.05).

## Results

### Effect of solid-fermentation with different grain substrates on the biomass and adenosine, HEA and polysaccharide concentration of *C. cicadae* NTTU 868 fruit bodies

**Fig. 1:**
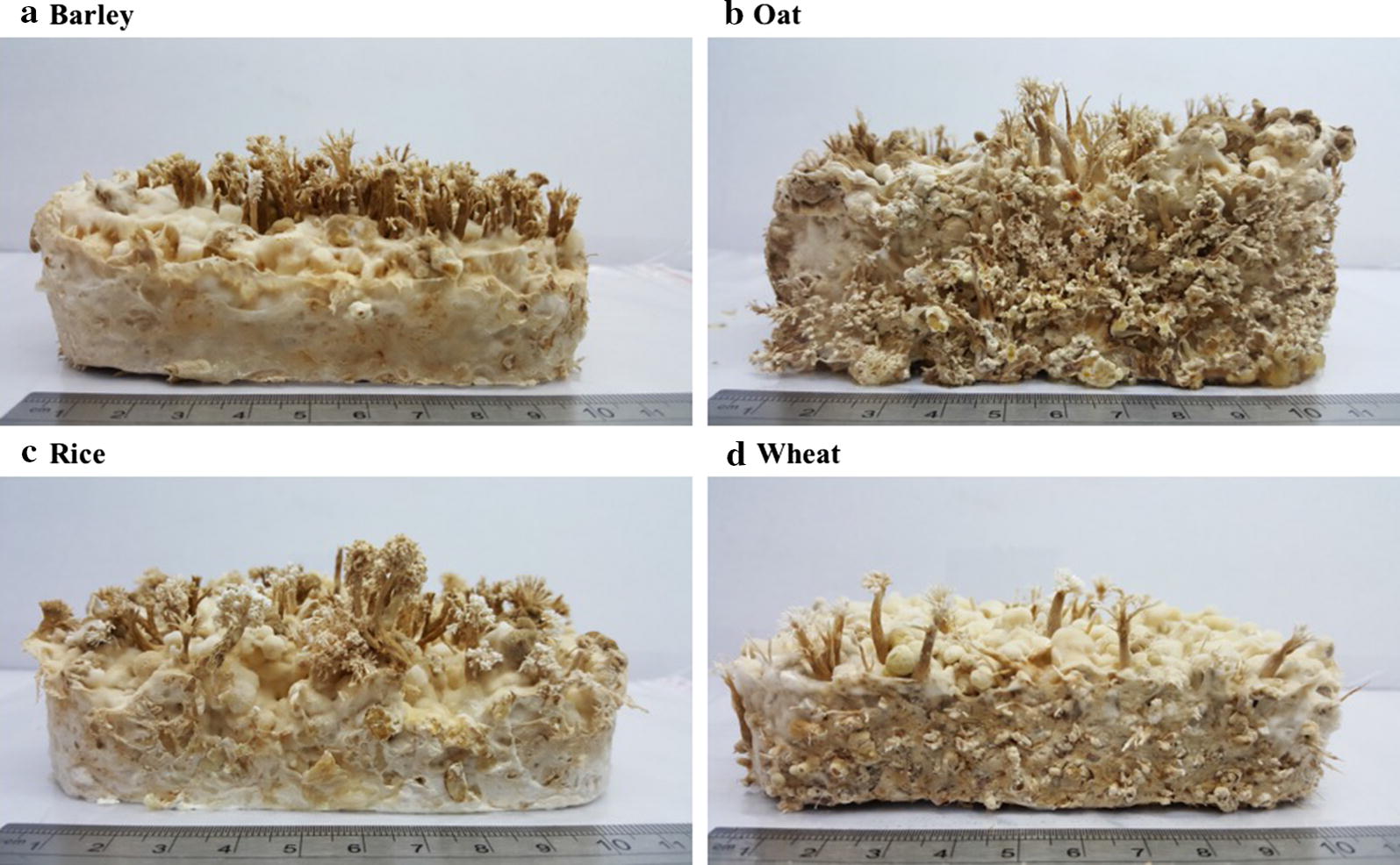
Fruit bodies of *C. cicadae* NTTU 868 fermented with different grain substrates **a** barley; **b** oat; **c** rice; **d** wheat

In this study, we evaluated the effect of different grain substrates on the production of fruit bodies and their bioactive compounds. The result of fruit bodies production was shown in Fig. 1. Using barley, oat, and rice as the substrates had higher production of fruit bodies than using wheat. *C. cicadae* NTTU 868 fruit bodies produced significantly higher adenosine concentration under oat substrate rather than under wheat substrate (*p *< 0.05) (Table 1). The *C. cicadae* NTTU 868 fruit bodies also produced significantly higher HEA concentration under barley, oat, and wheat substrates rather than under rice (*p *< 0.05). However, various grain substrates did not perform significantly difference on the concentration of polysaccharide produced by *C. cicadae* NTTU 868 fruit bodies (*p *> 0.05). As a result, the oat was regarded as the optimal substrate to produce higher adenosine and HEA concentrations of *C. cicadae* NTTU 868 fruit bodies.

**Table 1:** The biomass and adenosine, HEA and polysaccharide concentration in solid-fermented *C. cicadae* NTTU 868 fruit bodies with different grain substrates

Substrates	Biomass (g)	Adenosine (mg/kg biomass)	HEA (mg/kg biomass)	Polysaccharide (g/kg biomass)
Barley	2.56 ± 1.63^b^	893.5 ± 282.2^ab^	930.7 ± 24.5^b^	21.2 ± 2.5^a^
Oat	4.53 ± 1.93^bc^	1176.9 ± 120.6^b^	1034.2 ± 112.6^b^	24.4 ± 1.2^a^
Rice	5.44 ± 0.43^c^	851.0 ± 103.2^ab^	713.4 ± 87.4^a^	21.4 ± 2.2^a^
Wheat	0.35 ± 0.14^a^	730.4 ± 153.6^a^	1100.2 ± 124.2^b^	21.2 ± 0.4^a^

The data is presented as the mean ± SD (n = 3)

^a,b^Different letters indicate significantly different values according to a one-way ANOVA with Duncan’s multiple test (*p* < 0.05)

### Growth of submerged-fermented *C. cicadae* NTTU 868 mycelium and the change of adenosine and HEA concentration

**Fig. 2:**
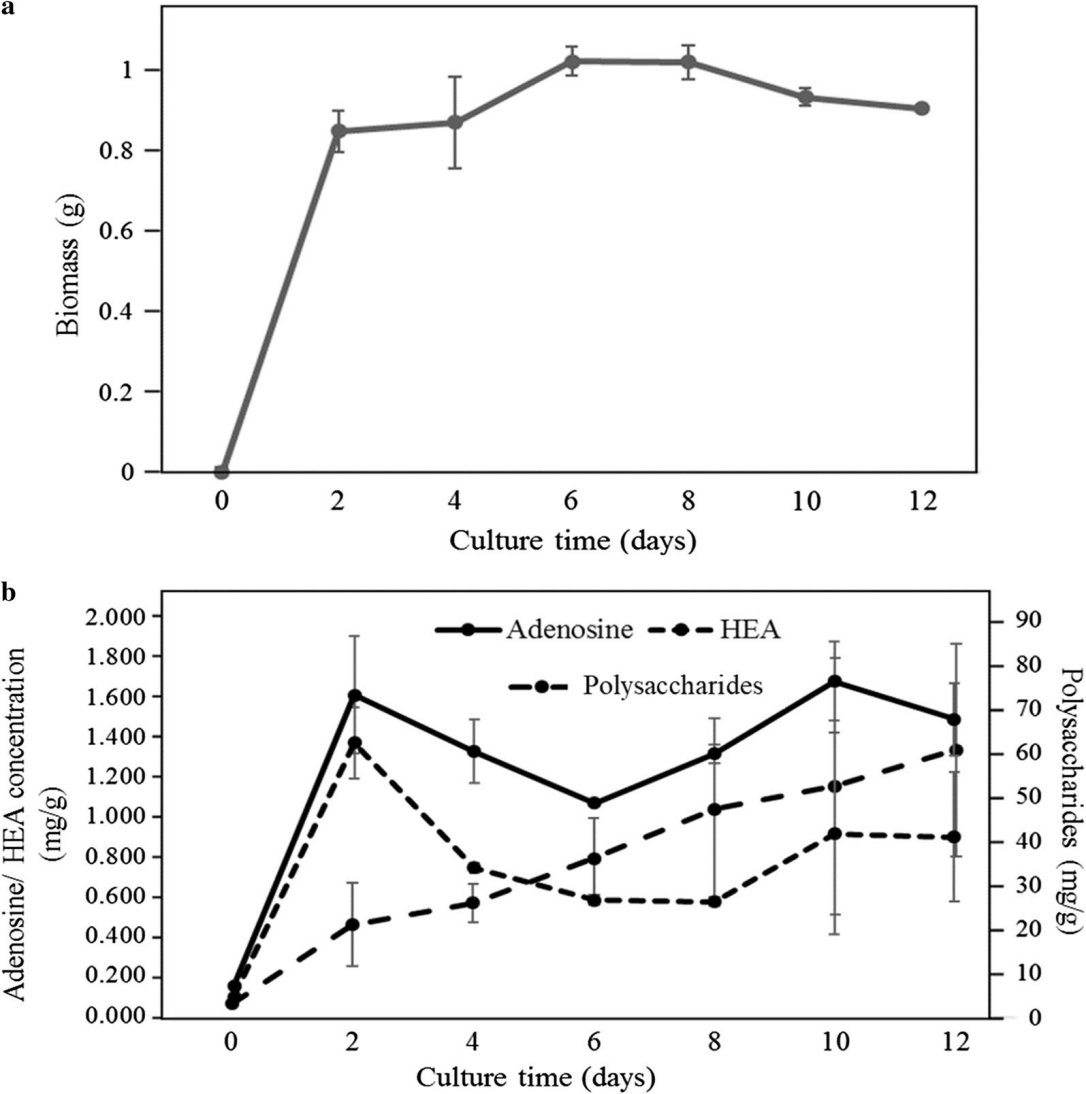
Growth curve of submerged-fermented *C. cicadae* NTTU 868 mycelium and the change of adenosine and HEA concentration: **a** dry mycelium biomass; **b** adenosine, HEA and polysaccharide concentration by using HPLC analysis. The data is presented as the mean ± SD (n = 3)

**Fig. 3:**
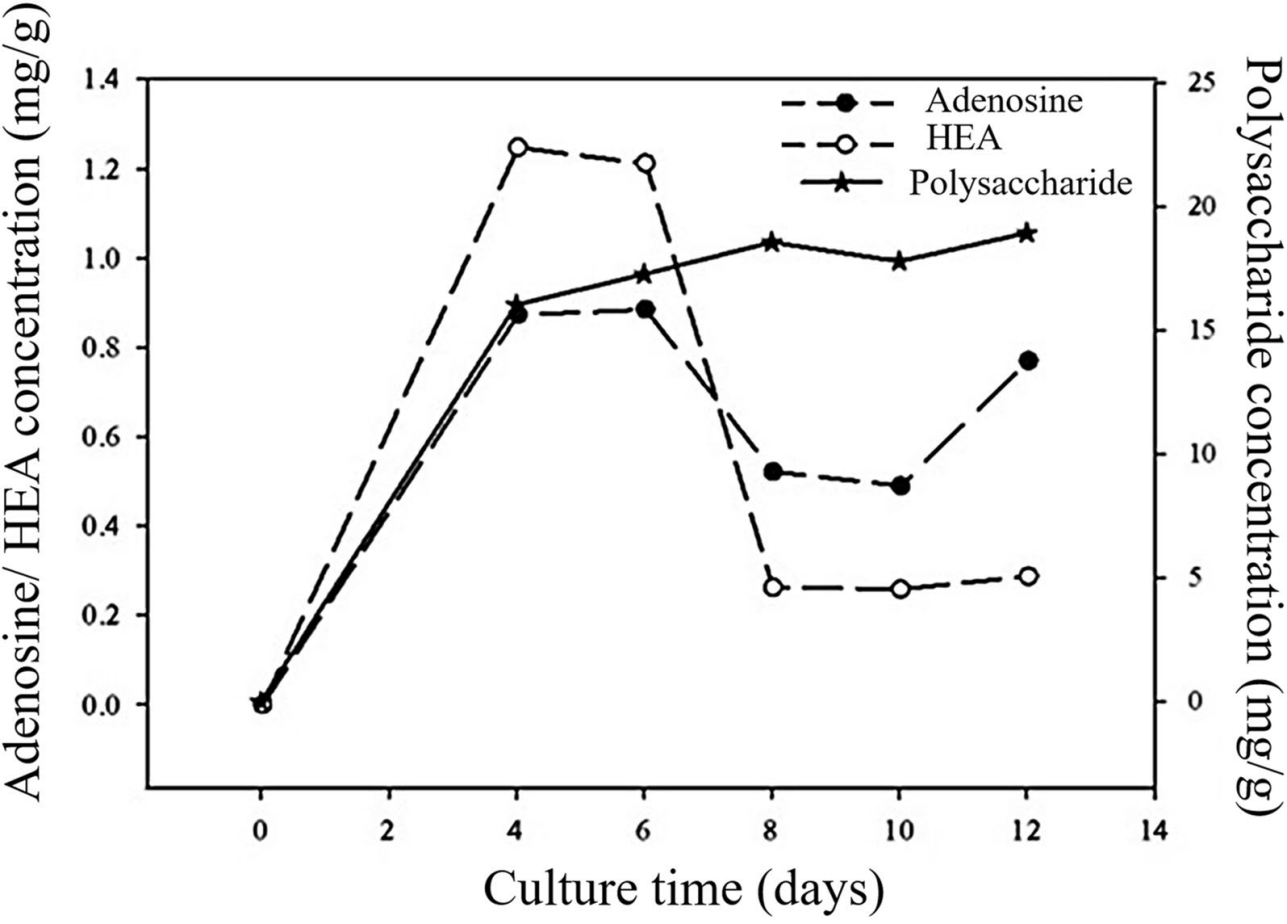
Time course of adenosine, HEA and polysaccharide contents under pilot-up fermentation of *C. cicadae* NTTU 868

We also investigated the growth curve of *C. cicadae* NTTU 868 mycelium. The growth curve is shown in Fig. 2a. The biomass can be rapidly increased before Day 2. The growth curve of biomass reached the stationary phase from Day 2 to Day 10. As shown in Fig. 2b, higher adenosine and HEA concentrations were observed on Day 2 and Day 10. The reason may be that the adenosine, a purine nucleoside, is an important material for the biomass growth and metabolism. However, adenosine and HEA concentrations decreased dramatically from Day 2 to Day 6, and then increased until Day 10. Furthermore, the fermentation capacity was scaled up in order to investigate whether adenosine, HEA and polysaccharide concentrations were stable using 1000-mL scale of submerged fermentation. As shown in Fig. 3, *C. cicadae* NTTU 868 mycelium had significantly higher adenosine and HEA concentrations on Day 4. However, adenosine and HEA concentrations decreased dramatically from Day 6 and then it slightly increased after Day 10. The production curve of polysaccharide in 1000-mL scale was similar to that in 100-mL scale, the polysaccharide production was increased with the increasing culture time.

### Effect of different basal mediums on adenosine, HEA and polysaccharide in 100-mL submerged-fermented *C. cicadae* NTTU868 mycelium

In order to investigate the culture condition of *C. cicadae* NTTU 868 mycelium, various grain powders such as the barley, oat, and rice powder (2%) were used as the culture substrate to produce mycelium respectively. As shown in Table 2, adenosine concentrations produced under barley, oat, and rice substrates on Day 10 were significantly higher than that on Day 2 (*p *< 0.05). The increased trend was also found on HEA concentrations produced under barley and oat substrates. However, we also compared adenosine and HEA concentrations of fermented products between grain powders and PDB. PDB had significantly greater adenosine and HEA concentrations than the other grain powders (*p *< 0.05). Furthermore, HEA concentration had reached the highest level on Day 2, which indicated that submerged culture with PDB can be used as a rapid fermentation mode for HEA production of *C. cicadae.*

**Table 2:** Effect of different basal mediums on adenosine, HEA and polysaccharide in 100-mL submerged-fermented *C. cicadae* NTTU868 mycelium

Substrates	Adenosine (mg/kg biomass)		HEA (mg/kg biomass)	
	Day 2	Day 10	Day 2	Day 10
Barley	75.8 ± 41.2^a^	442.4 ± 16.4^b^	45.1 ± 13.0^a^	114.0 ± 6.6^b^
Oat	102.9 ± 44.9^a^	529.3 ± 79.3^b^	3.9 ± 1.6^a^	102.8 ± 11.7^b^
Rice	17.5 ± 12.3^a^	225.5 ± 52.7^b^	1.2 ± 3.0^a^	9.2 ± 7.8^a^
PDB	1601.6 ± 295.7^a^	1669.3 ± 199.0^a^	1359.5 ± 176.8^a^	903.6 ± 509.2^b^

The data is presented as the mean ± SD (n = 3)

^a,b^Different letters indicate significantly different values according to a one-way ANOVA with Duncan’s multiple test (*p* < 0.05)

### Effect of carbon, nitrogen and mineral supplement on adenosine, HEA and polysaccharide contents of 100-mL submerged-fermented *C. cicadae* NTTU868 mycelium

To optimize the submerged culture condition of *C. cicadae* NTTU 868, 0.2% of carbon sources (dextrose, mannitol, or sucrose), 0.2% of nitrogen sources (monosodium glutamate, peptone or yeast extract) and 0.05% of mineral supplement (potassium chloride, magnesium chloride or ferric chloride) were added into the PDB, respectively. The result in Table 3 indicated that all of the additional carbon sources (dextrose, mannitol, sucrose) in the PDB performed no significant result on adenosine, HEA, and polysaccharide concentrations among each group (*p *> 0.05). Using peptone and yeast extract as additional sources of nitrogen source produced higher adenosine concentration than using MSG (*p *< 0.05). However, all of the additional nitrogen sources had no significant effect on HEA and polysaccharide concentration among each group (*p *> 0.05). The addition of ferric chloride may inhibit the growth of *C. cicadae* NTTU 868, resulting in significantly lower concentrations of adenosine, HEA and polysaccharide than potassium chloride and magnesium chloride (*p *< 0.05). The additional potassium chloride and magnesium chloride in the PDB was not able to increase adenosine concentration (*p *> 0.05). However, additional potassium chloride in the PDB contained higher concentrations of HEA than in magnesium chloride in the PDB (*p *< 0.05).

**Table 3:** Effect of carbon, nitrogen and mineral supplement on adenosine, HEA and polysaccharide contents of 100-mL submerged-fermented *C. cicadae* NTTU868 mycelium

Substrates	Adenosine (mg/kg biomass)	HEA (mg/kg biomass)	Polysaccharide (g/kg biomass)
Extra carbon sources			
Dextrose	1095 ± 16^a^	473 ± 7^a^	16.2 ± 0.9^a^
Mannitol	884 ± 356^a^	425 ± 57^a^	15.6 ± 0.8^a^
Sucrose	964 ± 274^a^	386 ± 82^b^	15.9 ± 0.9^a^
Extra nitrogen sources			
MSG	607 ± 46^a^	1335 ± 1230^a^	15.9 ± 1.7^a^
Peptone	1093 ± 22^b^	770 ± 57^a^	17.6 ± 1.6^a^
Yeast extract	1266 ± 339^b^	1171 ± 1315^a^	16.8 ± 1.1^a^
Extra mineral supplement			
KCl	1038 ± 49^b^	481 ± 118^c^	16.4 ± 0.7^b^
MgCl_2_	960 ± 143^b^	279 ± 6^b^	15.9 ± 0.6^ab^
FeCl_3_	344 ± 57^a^	109 ± 5^a^	15.0 ± 0.4^a^

The data is presented as the mean ± SD (n = 3)

*MSG* monosodium glutamate, *KCl* potassium chloride, *MgCl*_*2*_ magnesium chloride, *FeCl*_*3*_ ferric chloride

^a,b^Different letters indicate significantly different values according to a one-way ANOVA with Duncan’s multiple test (*p* < 0.05)

### Effect of different concentration of dextrose, yeast extract and KCl on adenosine, HEA and polysaccharide in 100-mL submerged-fermented *C. cicadae* NTTU868 mycelium

An additional 0.2% of dextrose in the PDB resulted in the *C. cicadae* NTTU 868 mycelium having higher adenosine and HEA content. We researched different concentrations (0%, 0.04%, 0.2% or 1.0%) of dextrose in the PDB to investigate the effect on the production of adenosine, HEA, and polysaccharide in the *C. cicadae* NTTU 868 mycelium. In Table 4, the culture medium without additional dextrose produced the highest HEA and polysaccharide concentrations in the *C. cicadae* NTTU 868 mycelium (*p *< 0.05). Therefore, additional dextrose was not able to increase the production of HEA and polysaccharide.

**Table 4:** Effect of different concentration of dextrose, yeast extract and KCl on adenosine, HEA and polysaccharide in 100-mL submerged-fermented *C. cicadae* NTTU868 mycelium

Sources	Adenosine (mg/kg biomass)	HEA (mg/kg biomass)	Polysaccharide (g/kg biomass)
Extra dextrose (%)			
0.00	1080 ± 56^b^	533 ± 33^c^	20.5 ± 1.1^b^
0.04	1020 ± 56^b^	472 ± 15^bc^	18.9 ± 2.0^ab^
0.20	964 ± 90^b^	432 ± 59^b^	17.6 ± 0.2^a^
1.00	656 ± 125^b^	337 ± 24^a^	19.4 ± 0.9^ab^
Extra yeast extract (%)			
0.00	1080 ± 56^a^	533 ± 33^a^	20.5 ± 1.1^a^
0.04	1134 ± 81^a^	544 ± 38^a^	21.3 ± 4.0^a^
0.20	1157 ± 376^a^	764 ± 120^b^	20.8 ± 2.5^a^
1.00	1169 ± 22^a^	634 ± 137^ab^	22.4 ± 6.1^a^
Extra KCl (%)			
0.00	1080 ± 56^c^	533 ± 33^b^	20.5 ± 1.1^a^
0.01	1035 ± 60^c^	479 ± 40^b^	21.2 ± 1.0^a^
0.05	841 ± 38^b^	474 ± 81^b^	20.0 ± 1.6^a^
0.25	670 ± 56^a^	356 ± 51^a^	21.6 ± 0.7^a^

The data is presented as the mean ± SD (n = 3)

*KCl* potassium chloride

^a,b,c^Different letters indicate significantly different values according to a one-way ANOVA with Duncan’s multiple test (*p* < 0.05)

According to Table 3, an additional 0.2% yeast extract in the PDB resulted in higher adenosine production in *C. cicadae* NTTU 868 mycelium. We compared different concentrations (0%, 0.04%, 0.2% or 1.0%) of yeast extract in the PDB and conducted research to find the best recipe for the culture medium. The result indicated that 0.2% of yeast extract in the PDB significantly increased the HEA production in *C. cicadae* NTTU 868 mycelium (*p *< 0.05). However, the productions of adenosine and polysaccharide in the *C. cicadae* NTTU 868 mycelium showed no significantly difference (*p *> 0.05) as using various concentrations of additional yeast extract.

Furthermore, an additional 0.05% of potassium chloride in the PDB produced higher levels of adenosine, HEA and polysaccharide in the *C. cicadae* NTTU 868 mycelium than when magnesium chloride and ferric chloride were used. However, lower concentrations of potassium chloride in the PDB produced significantly higher levels of adenosine and HEA in the *C. cicadae* NTTU 868 mycelium (*p *< 0.05). The levels of polysaccharide in the *C. cicadae* NTTU 868 mycelium had no significant change as using various concentrations (0%, 0.01%, 0.05% or 0.25%) of additional potassium chloride (*p *> 0.05).

### The difference of adenosine, HEA and polysaccharide production of *C. cicadae* NTTU 868 mycelium between different cultivation volume submerged fermentation

In the end, we compared the levels of adenosine, HEA and polysaccharide in two different culture scale (100-mL or 1500-mL) of cultured *C. cicadae* NTTU 868 mycelium. The result (Table 5) indicated that 100-mL of culture scale (with an additional 0.2% of yeast extract in the PDB) produced significantly higher levels of adenosine and polysaccharide than 1500-mL of the culture scale (*p *< 0.05). In contrast, 1500-mL of culture scale produced significantly higher levels of HEA than 100 mL of culture scale (*p *< 0.05). Therefore, submerged culture with PDB medium including additional 0.2% of yeast extract was proven to perform a rapid and high productivity culture mode for scale-up production of the functional compound HEA of *C. cicadae* NTTU 868.

**Table 5:** The difference of adenosine, HEA and polysaccharide production of *C. cicadae* NTTU 868 mycelium between different cultivation volume submerged fermentation

Culture scale (mL)	Adenosine (mg/kg biomass)	HEA (mg/kg biomass)	Polysaccharide (g/kg biomass)
100	1157 ± 376	764 ± 120	20.7 ± 2.5
1500	240 ± 31	1157 ± 449	14.3 ± 0.4

The data is presented as the mean ± SD (n = 3)

## Discussion

The wild *C. cicadae* fruit bodies usually grow in high humidity conditions on mountains between 80 and 500 m high. Because wild *C. cicadae* fruit bodies grow slowly and are easily infected by other moulds, the artificially-cultured *C. cicadae* fruit bodies have higher safety and more efficient to produce. In this study, *C. cicadae* NTTU 868 was a high production strain for HEA and polysaccharide productions. This study researched optimal solid fermentation conditions in order to develop the artificial culture technology. In previous research, it was shown that artificial-cultured *C. cicadae* fruit bodies grew in silkworms (Hu et al. 2009). The success rate of artificial-cultured *C. cicadae* fruit bodies is almost 100% using silkworm pupae, and about 70% using silkworm larvae. Another *C. cicadae* strain, *C. cicadae* MP12 was collected from the Shennongjia Mountains in mainland China. The artificial-cultured *C. cicadae* MP12 fruit bodies were successfully grown on *Cryptotympana atrata* pupae and a rice base medium (Wang et al. 2012). However, these materials are not easy to get, which will limit the scale-up production in the industrial development. In this study, we used barley, oat, rice and wheat as grain substrates to culture *C. cicadae* NTTU 868 fruit bodies. Using oat as the substrate was able to produce the highest content of fruit bodies than using the other substrates. In general, rice and wheat were used as the main grain substrates for the culture of *Cordyceps* species. However, *C. cicadae* NTTU 868 fruit bodies produced the lowest contents of HEA under the rice substrate. This study found that oat was the optimal solid substrate rather than wheat for the productions of fruit bodies, adenosine, and HEA. However, the culture time of solid fermentation was still over than 60 days.

Fermentation time and scale-up were both regarded as the important limitation factors for the development of fungi industry. Submerged culture is easy to scale-up, because it can be carried out by automatic production to achieve concatenation work, save time and improve efficiency. Therefore, this study further investigated the submerged culture of *C. cicadae* NTTU 868 mycelium. Mycelium produced by submerged culture and fruit bodies by solid culture had similar amounts of adenosine and HEA. However, the time of submerged culture was less than 10 days. Submerged fermentation produced the highest amounts of adenosine and HEA in *C. cicadae* NTTU 868 mycelium on day 2 and day 10. It also produced higher biomass of *C. cicadae* NTTU 868 mycelium. Therefore, submerged fermentation of *C. cicadae* NTTU 868 can easy scale up in the fermentation industry.

Although submerged culture was suitable technology to produce mycelium of *C. cicadae*, the culture medium should be a key factor for high production of HEA. Therefore, the previous studies also studied the effect of the medium compositions containing different carbon sources, nitrogen sources and mineral supplements on the growth and the production of bioactive compounds in *C. cicadae*. However, the previous study on the production of HEA was still rare, but we can understand the effect of medium composition in the previous studies of polysaccharides fermentation of *C. cicadae* or submerged fermentation of other *Cordyceps* species (Sharma et al. 2015a, b). The carbon sources including mannitol, sucrose and glucose increased the extracellular polysaccharide (EPS) and intracellular polysaccharide (IPS) production in the submerged culture of *C. cicadae* significantly (*p *< 0.05). Previous researches indicated that dextrose is the best carbon source for *Cordyceps* species (Sharma et al. 2015a, b). In this study, adding 0.2% by volume quantity of dextrose in the PDB resulted in higher concentrations of adenosine and HEA in the *C. cicadae* NTTU 868 mycelium than using mannitol or sucrose. In the previous study, yeast extract and peptone both produced higher amounts of EPS and IPS in the *C. cicadae* mycelium than other nitrogen sources (Sharma et al. 2015b). In this study, 0.2% of yeast extract was still the optimal additional nitrogen source in the PDB for increasing the concentration of adenosine and HEA. Above results were also similar to the results of other studies on another *C. cicadae* strain and *C. sinensis* (Dong and Yao 2005). Therefore, yeast extract was a well nitrogen source for the culture of many *Cordyceps* species as well as the production of HEA of *C. cicadae.* Ions and salts also affected the growth and metabolism of fungi. The mineral sources, monopotassium phosphate, cobalt(II) chloride hexahydrate and iron(II) sulfate heptahydrate, in the culture medium increased the concentrations of EPS and IPS in the *C. cicadae* mycelium (Sharma et al. 2015b). Potassium chloride has been observed as being the optimal mineral source to produce higher concentrations of adenosine in *C. militaris* (Hung et al. 2015) and higher concentration of EPS and IPS in another *C. cicadae* strain and *C. sinensis* (Dong and Yao 2005; Sharma et al. 2015a). This study also observed that additional potassium chloride in PDB was able to produce higher productions of adenosine and HEA in the *C. cicadae* NTTU 868 mycelium.

Adenosine, HEA and polysaccharide performed different production curves during the fermentation of *C. cicadae* NTTU 868. Furthermore, *C. cicadae* NTTU 868 performed different growth curve in 100-mL and 1500-mL fermentation scales. The highest levels of concentration of adenosine and HEA were observed on Day 2 and Day 4 in 100-mL and 1500-mL fermentation scale, respectively. Polysaccharide production can be increased with the fermentation time in 100-mL or 1500-mL fermentation scales. However, Day 4–6 can be regarded as the optimal fermentation time for the production of adenosine, HEA and polysaccharide in the 1500-mL submerged fermentation scale. In conclusion, solid and submerged fermentation can produce similar concentrations of adenosine, HEA and polysaccharide. Adding 0.2% of yeast extract in the PDB, at 24 °C for 10 days created the most suitable fermentation condition for the *C. cicadae* NTTU 868 mycelium. However, submerged fermentation is more efficient for adenosine and HEA production than using solid-fermentation. Importantly, it can be easily scaled-up in the fermentation industry to develop medical potential in the industry.

## Data Availability

Not applicable.
